# Ovarian mucinous borderline tumor accompanied by LGESS with myxoid change: a case report and literature review

**DOI:** 10.1186/s40001-017-0295-4

**Published:** 2017-12-02

**Authors:** Wen Wang, Yalin Zhuang, Feng Zhou, Lili Huang

**Affiliations:** 1 Department of Obstetrics and Gynecology, Women’s Hospital, School of Medicine, Zhejiang University, Hangzhou, Zhejiang China; 2 Department of Pathology, Women’s Hospital, School of Medicine, Zhejiang University, 1 Xueshi Road, Hangzhou, 310006 Zhejiang People’s Republic of China

**Keywords:** Mucinous borderline tumor, Mural nodules, LGESS, Myxoid change

## Abstract

**Objectives:**

To report an extremely rare case of ovarian borderline mucinous cystic tumor accompanied by low-grade endometrial stromal sarcoma (LGESS) with myxoid change.

**Case presentation:**

A 42-year-old woman complained of lower left abdominal fullness. Her serum carcinoembryonic antigen, cancer antigen (CA) 125, and CA19-9 levels were normal. Magnetic resonance imaging showed a 10-cm cystic mass with a 5-cm nodule in its wall, and a laparoscopy indicated a cystic mass at the left adnexa. Histology indicated a cystic lesion consisting of proliferative gastrointestinal-type epithelium; the mural nodule had a characteristic of striking myxoid change, preservation of arteriolar pattern, and a “tongue-like” infiltration.

**Conclusions:**

The diagnosis of ovarian mucinous borderline tumor accompanied by LGESS with myxoid change was appropriate.

## Background

Mucinous cystic tumors of the ovary, whether benign, borderline, or malignant, may be associated with mural nodules of various types, including sarcomas, sarcoma-like mural nodules, foci of anaplastic carcinoma, carcinosarcoma, mixed nodules, and leiomyomas [1, 2]. The subject of this report is a mural nodule with features of a low-grade endometrial stromal sarcoma (LGESS) with myxoid change arising in an ovarian mucinous borderline tumor. To our knowledge, there are no reports of mucinous ovarian tumors accompanied by LGESS with myxoid change.

## Case report

A 42-year-old woman complained of lower left abdominal fullness. Her serum carcinoembryonic antigen (CEA), cancer antigen (CA) 125, and CA19-9 levels were within normal limits. Magnetic resonance imaging (MRI) showed a 10-cm cystic mass at the pelvic cavity with a 5-cm nodule in its wall. The laparoscopy showed a 10-cm cystic mass at the left adnexa. The right ovary and uterus were grossly normal. The patient underwent left salpingo-oophorectomy and adhesiolysis. The patient was symptom free 10 months after surgery.

**Fig. 1:**
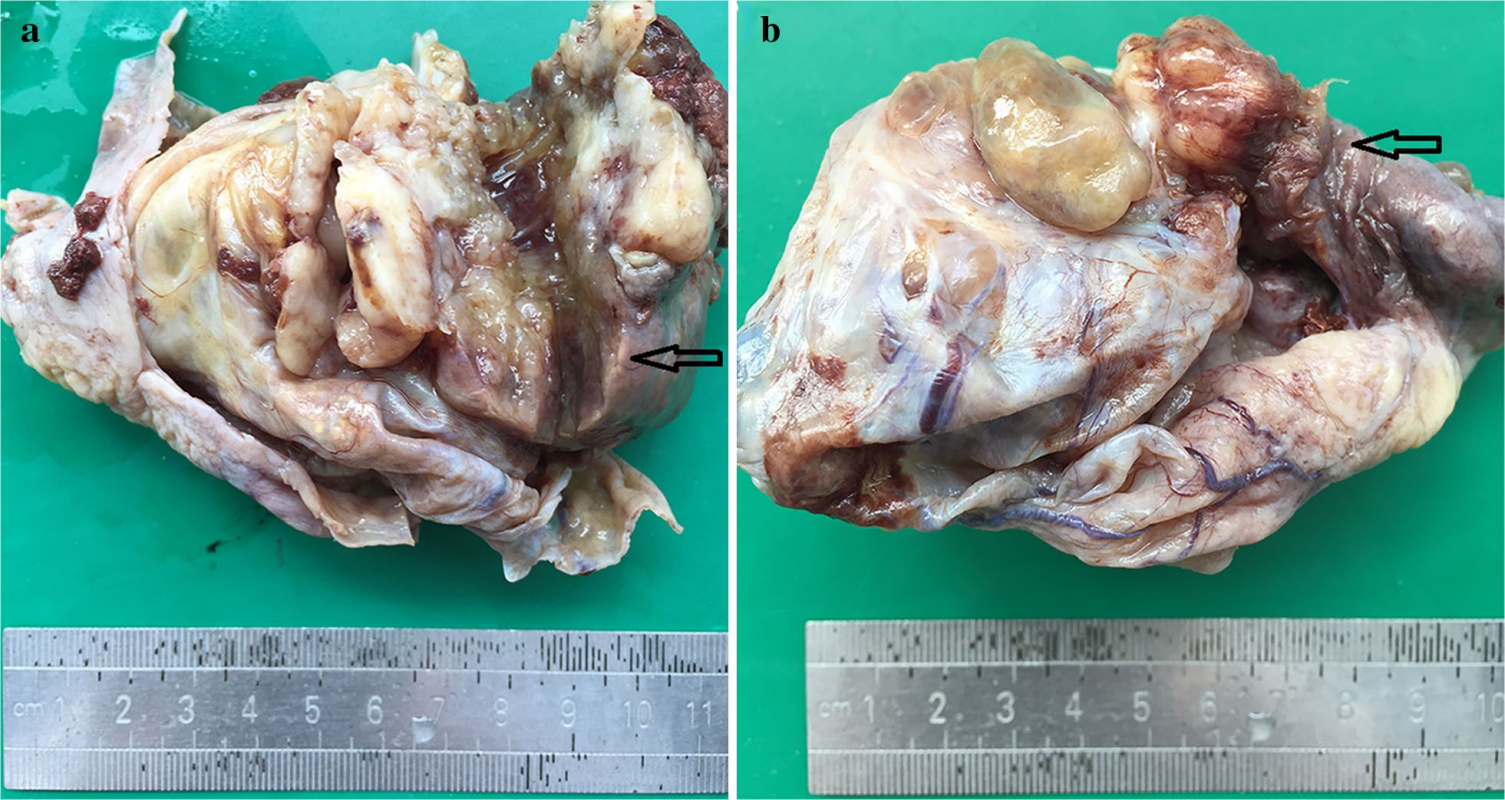
Gross specimen of ovarian cystic mass with mural nodule: a solid mural nodule protruding into the cyst indicated with black arrows (**a**). Penetrating the surface of the cyst and invading to the tubal fimbria indicated with black arrows (**b**)

On macroscopic examination, the cyst measured 10 cm in maximal diameter and had a thickness of 0.2–0.5 cm. This cystic tumor focally adhered to the peritoneum. The inner side was slightly uneven and attached with clot-like material in some area. The cyst had a solid mural nodule protruding from the wall into the lumen measuring up to 5 × 4 × 4 cm in size (Fig. 1a). The mass was found penetrating the surface of the cyst and invading to the tubal fimbria. The cut surface was fleshy and gray with the area of hemorrhage and necrosis (Fig. 1b).

**Fig. 2:**
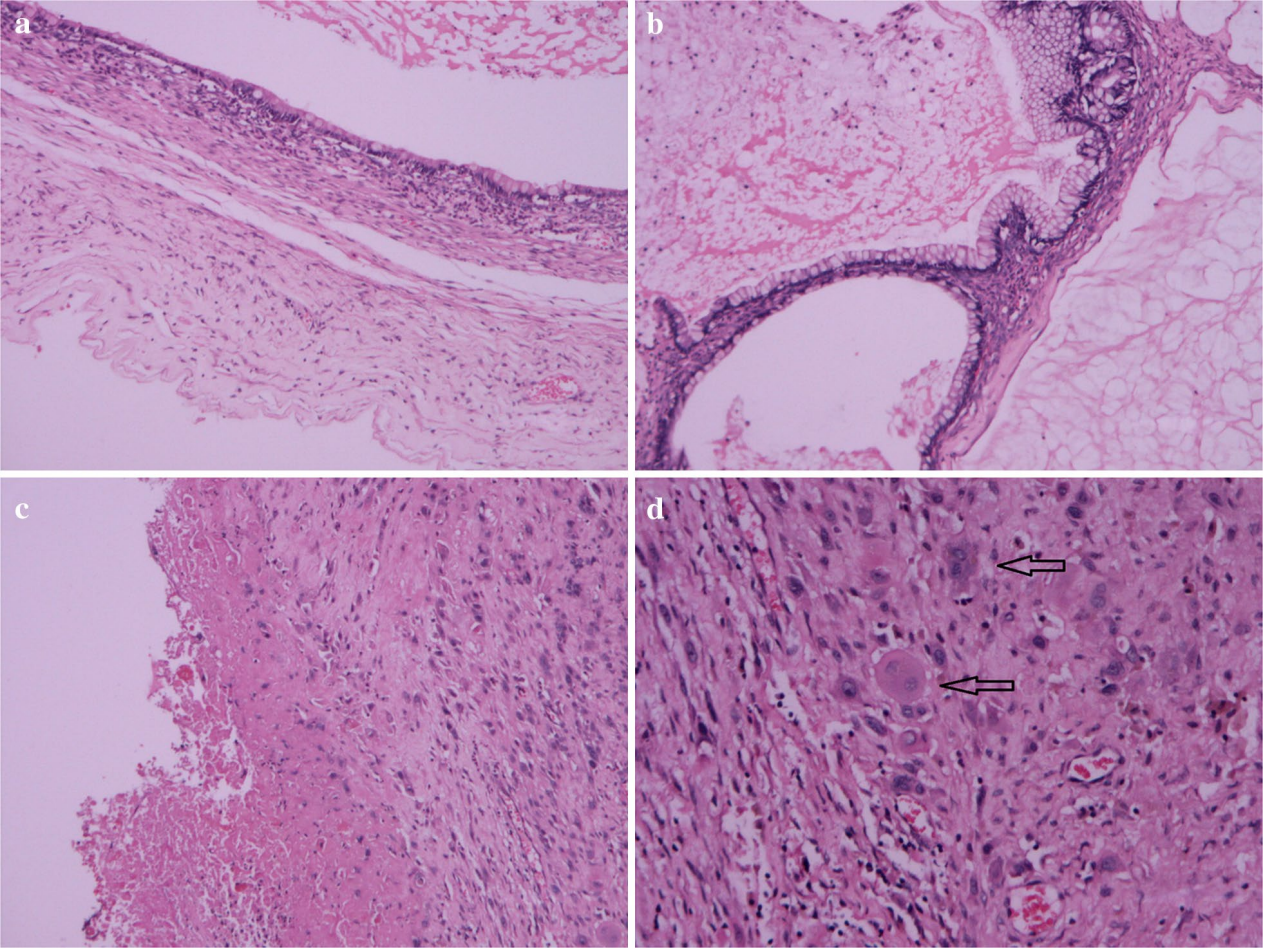
Histological findings (H&E). **a** Ovarian mucinous cystadenoma (×10). **b** Borderline malignant mucinous cystadenoma and acellular pools of mucin (×10). **c** Old hemorrhage in the cystic wall (×10). **d** Proliferation of large mononucleated cells and scattered multinucleated giant cells indicated with black arrows (×20)

Microscopic observation showed that the cystic walls were lined mainly by single-layered tall mucinous columnar epithelium (Fig. 2a). Some epithelium of cystic walls showed slightly atypical proliferation with glandular budding and stratification. The cells exhibited mild to moderate nuclear enlargement and hyperchromasia. Acellular pools of mucin in the stroma were present in some areas (Fig. 2b). Old hemorrhage can be found in the cystic wall (Fig. 2c). In addition, there was a proliferation of large mononucleated cells and scattered multinucleated giant cells in an area of the cyst wall without forming a true nodule (Fig. 2d).

**Fig. 3:**
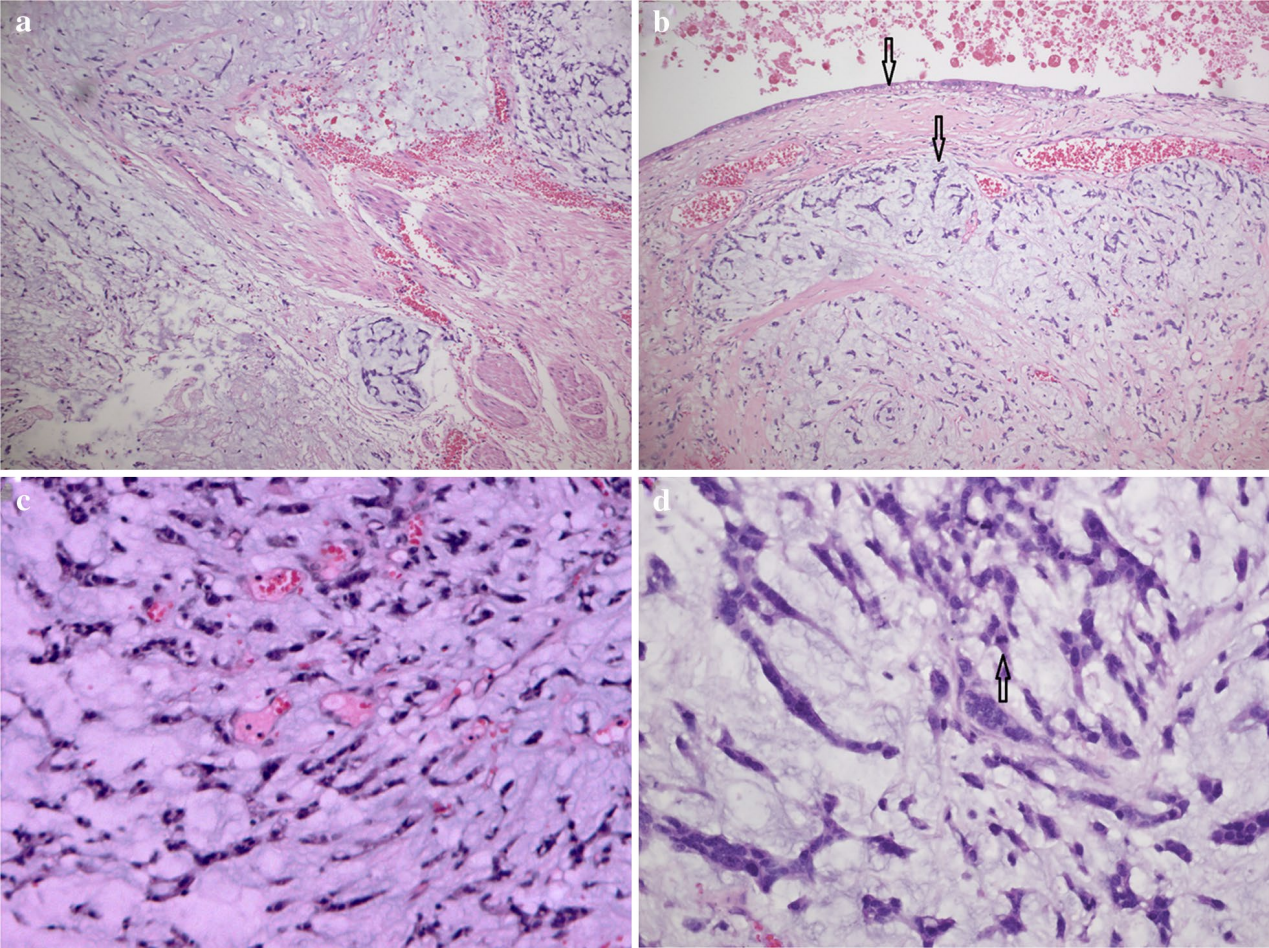
Histological findings (H&E). **a** Tongue-like processes extending into the edematous stroma (×10). **b** Sharp demarcation between epithelial and sarcomatous components indicated with black arrows (×10). **c** Preservation of arteriolar pattern (×20). **d** Nuclear atypia and the mitosis indicated with black arrows (×40)

A 5-cm mural nodule was identified in the hysterectomy specimen protruding into the cystic cavity, extending onto the serosal, and involving the tubal fimbria. The mural nodule had a characteristic of striking myxoid change, preservation of arteriolar pattern, and a “tonguelike” infiltration (Fig. 3a). There was a sharp demarcation between epithelial and sarcomatous components (Fig. 3b). The mural nodule consisted of sheets of small cells with oval to spindle nuclei, whorling around spiral arteriole-like vessels (Fig. 3c). There was mild to moderate nuclear atypia and the mitosis was 4/10 HPF (Fig. 3d).

**Fig. 4:**
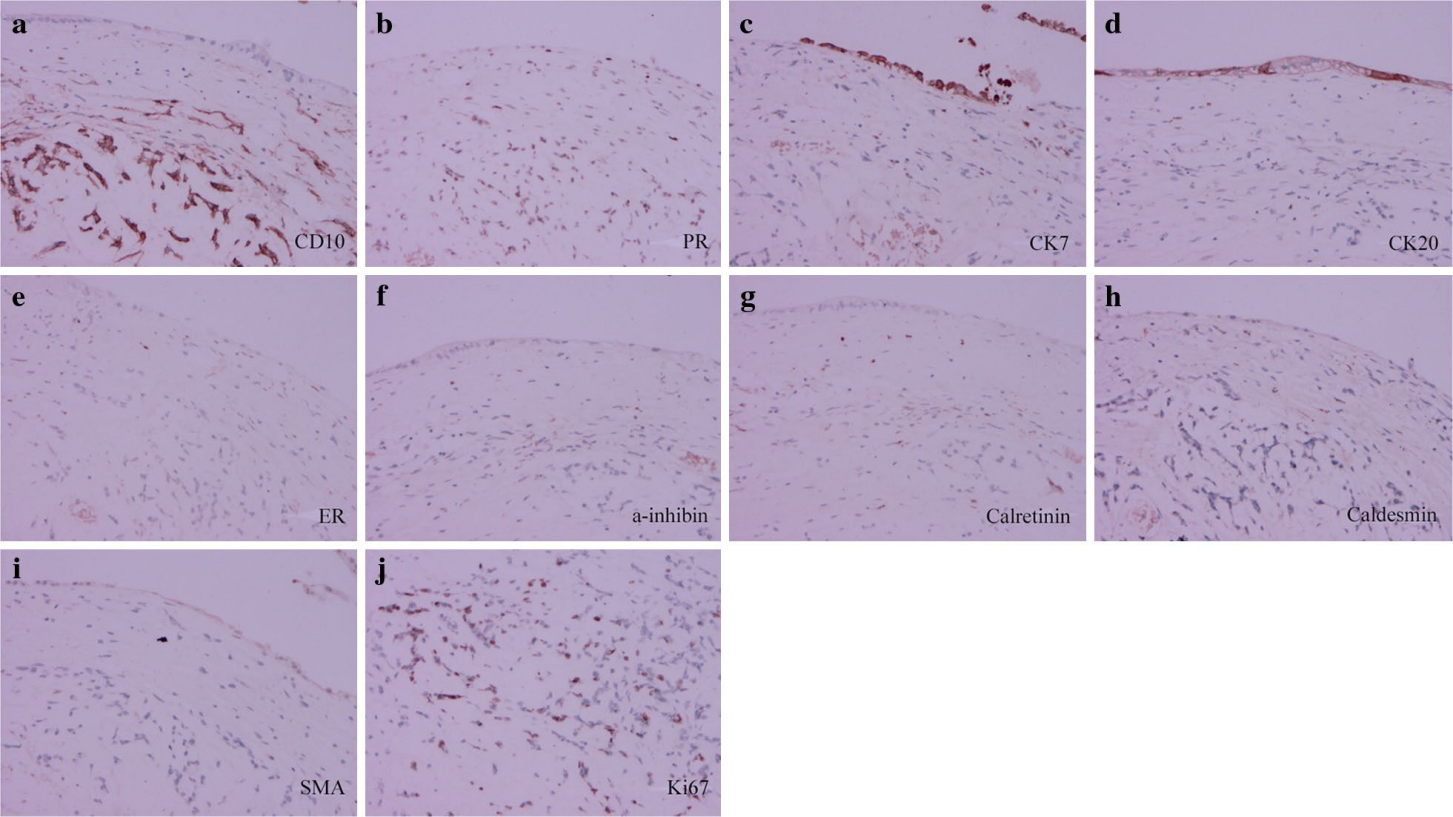
Immunochemical photograph of the ovarian tumors: CD10 (**a**, ×20), PR (**b**, ×20), CK7 (**c**, ×20), CK20 (**d**, ×20), ER (**e**, ×20), a-inhibin (**f**, ×20) calretinin (**g**, ×20), caldesmon (**h**, ×20), SMA (**i**, ×20), and Ki67 (**j**, ×20)

Immunohistochemical staining is shown in Fig. 4. The tumor cells of the mural nodule were strongly positive for cluster of differentiation 10 (CD10) and progesterone receptor (PR) and was negative for cytokeratin 7 (CK7), CK20, estrogen receptor (ER), a-inhibin, calretinin, caldesmon, and smooth muscle actin (SMA), which is a typical feature of endometrial stromal sarcoma. The tumor cells had a high index of Ki67. The epithelium of cystic wall was locally positive for CK7 and CK20.

Taking into account the above features, we made the diagnosis as ovarian mucinous borderline tumor accompanied by LGESS with myxoid change involving the tubal fimbria.

## Discussion

In 1979, Prat and Scully [3] described in detail the clinicopathologic features of mural nodules emphasizing their broad morphologic spectrum and varying biological behavior. Since then, the scope of ovarian mural nodules has been classified into sarcoma-like, sarcoma, and anaplastic carcinoma [4]. The present case is quite interesting because of the presence of LGESS with myxoid change.

Mural nodules can develop in benign, borderline, or malignant ovarian tumors. These mural nodules are seen as solid lesions on the wall of the tumor or protrude into the cyst lumen. The diagnosis of mural nodules in the residual ovary is based on the size and characteristic gross and microscopic features [5, 6]. The benign and reactive mural nodules, also called sarcoma-like mural nodules (SLMNs), occur in younger females and are usually sharply demarcated small lesions. Malignant mural nodules are large solid tumors; as sarcoma and anaplastic carcinomas, they tend to occur in older females.

A review of the literature revealed only five mucinous ovarian tumors with sarcomas, with most of the mural nodules measured > 5 cm in the greatest dimension. In these five cases, they were fibrosarcomatous mural nodule in an ovarian mucinous cystadenoma [7], fibrosarcoma arising in a mucinous cystadenocarcinoma [8], fibrosarcoma associated with a mucinous cystadenoma [9], undifferentiated sarcoma in a mucinous cystadenocarcinoma [9], and rhabdomyosarcoma coexistent with ovarian mucinous cystadenocarcinoma [10], respectively. Ovarian mucinous borderline tumor accompanied by LGESS with myxoid change in our case was rather unique. The clinical and pathological features are summarized in Table 1.

**Table 1:** Clinical and pathological features of mucinous ovarian tumors with sarcomas

Authors [reference]	Age	Symptom	Figo stage	No. of nodules	Size (cm)	Mural nodule	Epithelial ovarian tumor	Treatment	Follow-up
De Nictolis et al. [7]	na	na	na	na	na	Fibrosarcoma	Mucinous cystadenoma	na	na
Rahilly et al. [8]	69	Lower abdominal pain	IIA	One	na	Fibrosarcoma	Mucinous cystadenocarcinoma	TAH-BSO + appendicectomy + CTX	ned at 12 month
Scully et al. [9]	61	Abdominal swelling	IA	One	10	Fibrosarcoma	Mucinous cystadenoma	TAH-BSO + RT	Died at 18 month
	49	Abdominal swelling	IVB	One	7	Undifferentiated sarcoma	Mucinous cystadenocarcinoma	BSO + OT + periaortic biopsy	Died at 1 week
Tsujimura et al. [10]	57	No obvious symptoms	IA	One	15	Rhabdomyosarcoma	Mucinous cystadenocarcinoma	TAH-BSO + CTX	ned at 3 month
This work	42	Abdominal fullness	IIA	One	5	LGESS with myxoid change	Mucinous borderline tumor	UA	ned at 10 month

ned, no evidence of disease; na, not available; TAH-BSO, tota abdominal hysterectomy and bilateral salpingo-oophorectomy; OT, omentectomy; SH, subtotal hysterectomy; UA, unilateral adnexectomy; RT, radiation therapy; CTX, chemotherapy

The differential diagnosis for extrauterine LGESS depends on the location of the tumor. In case of ovarian location, ovarian sex cord–stromal tumors should be excluded. The pathological and morphological characteristics and immunohistochemical findings in the present case do not favor ovarian sex cord–stromal tumors. LGESS with myxoid change can be confused with myxoid smooth muscle lesions, but the “tonguelike” infiltration and small arterioles, along with the CD10 immunostaining and muscle marker negativity, will usually resolve the diagnosis.

Tumors with a combination of different histology are divided into two clinicopathologic groups, collision or composite tumors [11]. The collision tumor has more than two juxtaposed masses and each mass displays a different histology. In contrast, the intermingling of more than two different components in one tumor mass is designated as a composite tumor. The well-known example is a malignant mixed Müllerian tumor (MMMT). It was noted that there was a sharp demarcation between epithelial and sarcomatous components in this case.

In this case, there was a region of proliferation of large mononucleated cells just beneath the epithelium without forming a true nodule, which may help explain the occurrence of malignant mural nodules. We hypothesize the forming process of this sarcoma as follows. The submesothelial mesenchymal cells reacted to the intramural hemorrhage or the mucinous content of the cyst, which eventually became a hyperplastic nodule, probably corresponding to the earlier stage of the SLMNs. Then the proliferation of mesenchymal cells resulted in the sustained expansion of the lesion. The nodule enlarged gradually and became SLMNs. Some of the SLMNs could possibly evolve into a true sarcoma with the proliferation and differentiation of mesenchymal cells.

## Conclusions

We have presented a rare case of sarcoma-like mural nodule. Based on the ample evidence of the previous reports, and the immunohistochemical analysis of our case, we believe that this is a collision tumor with two different elements and may be formed by SLMN malignant change. Nevertheless, further molecular and genetic evidence is needed to support such a conclusion.

## Abbreviations

LGESS: low-grade endometrial stromal sarcoma
CEA: carcinoembryonic antigen
CA125: cancer antigen 125
CA19-9: cancer antigen 19-9
MRI: magnetic resonance imaging
CD10: cluster of differentiation 10
PR: progesterone receptor
CK7: cytokeratin 7
CK20: cytokeratin 20
ER: estrogen receptor
SMA: smooth muscle actin
SLMN: sarcoma-like mural nodules
MMMT: malignant mixed Müllerian tumor
